# Benthic Microbial Community Features and Environmental Correlates in the Northwest Pacific Polymetallic Nodule Field, with Comparative Analysis Across the Pacific

**DOI:** 10.3390/microorganisms14010103

**Published:** 2026-01-03

**Authors:** Ziyu Li, Juan Yang, Xuebao He, Ziyu Zhao, Jianxin Xia

**Affiliations:** 1 School of Ocean Sciences, China University of Geosciences (Beijing), Beijing 100083, China; 2011230027@email.cugb.edu.cn (Z.L.); yangjuan@cugb.edu.cn (J.Y.);; 2 Key Laboratory of Polar Geology and Marine Mineral Resources, Ministry of Education, Beijing 100083, China; 3 Third Institute of Oceanography, Ministry of Natural Resources, Xiamen 361005, China

**Keywords:** deep-sea microorganisms, environmental correlations, regional comparison, co-occurrence networks

## Abstract

Microorganisms, as the foundation of deep-sea ecosystems, are crucial for maintaining the structure and stability of polymetallic nodule field environments. To investigate the community structure and distributional patterns of benthic microorganisms in such environments, this study used high-throughput sequencing to analyze the composition, diversity, and environmental correlations of bacteria, archaea, and fungi in the BPC (Beijing Pioneer Hi-tech Development Corporation Ltd., Beijing, China). Furthermore, microbial communities from BPC were compared with those from UK-1 (UK Seabed Resources, Southampton, UK) in terms of community structure and co-occurrence network characteristics. The results revealed that in the BPC, the bacterial communities were dominated by Proteobacteria and Chloroflexi, while Crenarchaeota represented the overwhelmingly dominant group. Fungal communities were primarily composed of Ascomycota and Basidiomycota. Correlation Analysis suggested that water depth, TOC (Total organic carbon), TN (Total nitrogen), and δ^15^N emerged as the key environmental drivers of microbial community variation. Comparative analysis showed microbial groups exhibited certain similarities but also some differences at the phylum, class, and order levels, with the differences becoming increasingly pronounced at finer taxonomic resolutions between BPC and UK-1. Co-occurrence network analyses indicated the microbial networks with higher density and node connectivity in the BPC, whereas the UK-1 exhibited greater modularity and clustering coefficients. Microbial interactions were weaker in the UK-1, but its resilience to benthic disturbance was expected to be higher than in the BPC.

## 1. Introduction

Polymetallic nodules, which are patchily distributed on deep-sea plains below the carbonate compensation depth (CCD), are enriched in manganese and iron, as well as economically valuable metals such as copper, nickel, and cobalt [1,2,3]. They are usually found covering the seafloor surface or embedded in the upper 5–10 cm layer of the bottom sediment [4,5,6]. As the deep sea hosts vast mineral resources and represents a critical alternative to terrestrial reserves, the commercial extraction of polymetallic nodules is anticipated to commence in the near future [7]. However, these metal-rich nodules not only represent valuable mineral resources, but also create heterogeneous microhabitats that may profoundly influence benthic microbial communities and associated biogeochemical processes [8].

Microorganisms constitute the largest biomass reservoir inhabiting the abyssal plains and underlying seafloor [9]. To date, research on microorganisms in deep-sea polymetallic nodule mining areas has primarily focused on the community structure and diversity of bacteria and archaea in the Clarion-Clipperton Zone (CCZ) of the eastern Pacific. Wear et al. [10] collected, processed, and synthesized data from multiple mining areas in the eastern CCZ, and demonstrated that spatial variability of bacterial and archaeal communities in benthic sediments is primarily attributed to differences in relative abundance rather than by shifts in dominant taxa. Moreover, they showed that the rate of spatial variation can be non-linear even across relatively short geographic distances.

In the CCZ, bacterial communities in sediments are phylogenetically diverse, dominated by Proteobacteria, Acidobacteria, and Planctomycete, whereas other bacterial groups occurred at much lower relative abundances [10,11,12,13,14,15,16]. In the western Pacific, Wang et al. [17] reported that bacterial communities were dominated by Proteobacteria and the Chloroflexi. Archaeal taxonomic diversity is generally lower than that of bacteria when compared within the same habitat [18]. Across most mining areas, Thaumarchaeota was the most enriched group within archaeal communities [10,15,19]. Although archaea account for only 19–36% of prokaryotes [15], they predominantly represent a vital source of extracellular enzymes and biocatalysts [20], thereby playing crucial roles in global carbon and nitrogen cycling. In particular, key processes such as methanogenesis, anaerobic methane oxidation, and ammonia oxidation to nitrite—central to carbon and nitrogen cycling—are carried out exclusively by archaea [21,22,23]. In contrast, Fungi, serving as crucial connectors between prokaryotes and eukaryotes in deep-sea sediment microbial communities, remain largely unexplored in polymetallic nodule fields. Previous studies indicate that bacteria are 1.4–5 times more efficient than fungi in utilizing simple organic compounds as substrates, whereas fungi outcompete bacteria by 1.1–4.1 times when metabolizing complex compounds [24]. Specific deep-sea fungi, predominantly classified as Ascomycota and Basidiomycota from the western Pacific, have been shown to produce diverse bioactive compounds and play a key role in N_2_O production during denitrification [25,26].

Large-scale surveys in the eastern CCZ also demonstrated that microhabitat (i.e., nodule or sediment) seemed to be a major factor influencing microbial community composition, rather than sampling locations or distances between fields [11]. In the extreme and highly complex environmental conditions of deep-sea ecosystems, microorganisms play crucial and diverse roles in energy acquisition and nutrient metabolism [27], making them highly sensitive to environmental variation. Yang et al. [28] indicate that sediment microbial communities are strongly shaped by environmental factors, particularly sediment geochemistry, such as ammonia nitrogen and total nitrogen, as well as overlying water properties including pH, temperature, electrical conductivity, and total dissolved solids.

Mining-induced alterations of microbial communities are expected to profoundly affect deep-sea benthic ecosystems. Previous studies in the CCZ have provided detailed characterizations of bacteria, archaea, and fungi individually; few have systematically examined their relationships with environmental variables within a unified analytical framework. However, the greater water depths, lower nodule coverage, and reduced nutrient availability characterizing western Pacific polymetallic nodule fields relative to the CCZ may lead to fundamentally different microbial community responses to mining disturbances [29,30,31]. To assess whether these findings are applicable under large-scale environmental differences between fields, traditional microbial indicators alone often exhibit high variability and limited resolution, thereby constraining the detection of ecological effects associated with subtle disturbances [32]. Further investigation of complex co-occurrence networks is critical for understanding microbial survival, adaptation, and the establishment of their functional roles in the environment [33], as key network properties can respond to subtle variability by 10–50% [34]. Du et al. [35] demonstrated that the stability of these co-occurrence networks among microorganisms is essential for maintaining ecosystem resilience. Collectively, these considerations highlight the need for integrated cross-domain, cross-regional analyses to clarify microbial biogeography, ecosystem resilience, and mining impacts.

Based on these environmental characteristics, comparing microbial communities between the eastern and western Pacific, with consideration of multi-domain microbial interactions and environmental variables, provides an effective framework for assessing how large-scale environmental differences influence community structure and their responses to mining-induced alterations. To address these underexplored aspects in the nodule field, this study conducted an integrated assessment of bacterial, archaeal, and fungal communities in Block M2 of the Beijing Pioneer Hi-tech Development Corporation (BPC) contract area in the western Pacific, incorporating environmental factors, using a standardized high-throughput sequencing and analytical pipeline. By reanalyzing the UK Seabed Resources (UK-1) contract area dataset from the eastern Pacific using a comparable methodological framework and updated taxonomic databases, robust comparisons between the UK-1 and BPC were achieved at both the community structure and co-occurrence network levels. This study specifically aimed to investigate as follows: (1) characterize the dominant microbial taxa and overall diversity in the BPC, (2) examine the correlations between different microbial groups and environmental factors, (3) compare the community structure of microbial taxa across different taxonomic levels between the two nodule fields, and (4) assess differences in stability between the two regions based on intergroup interactions within co-occurrence networks. This study provides a more comprehensive perspective on deep-sea microbial biogeography and ecosystem resilience than previously available. It evaluates whether deep-sea microbial communities exhibit region-specific characteristics or shared biogeographic patterns, thereby offering critical insights into their resilience, functional potential, and ecological vulnerability under future mining disturbances.

## 2. Materials and Methods

### 2.1. Sample Collection

**Figure 1:**
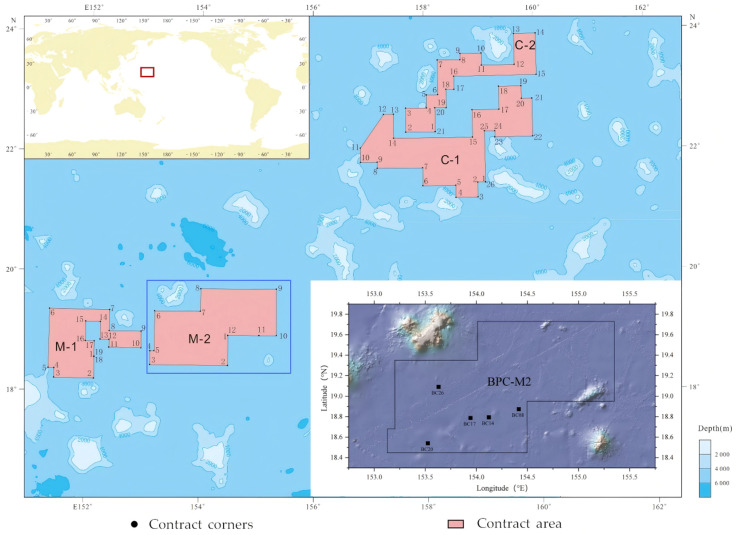
Sampling Station Distribution in the M2 Field, Western Pacific. The base map was created using data from the International Seabed Authority (https://www.isa.org.jm, accessed on 20 June 2025).

Sediment samples were collected during Cruise DY81 of the R/V *Dayang Yihao* in 2023 centered within the M2 region of the polymetallic nodule field of BPC in the northwestern Pacific Ocean (Figure 1). The study area was situated between 153–155° E and 18–19° N, with water depths ranging from 5500 to 5750 m. Sediment samples were retrieved using a box corer . Prior to deployment, the box corer and all sampling tools were thoroughly rinsed with deionized water, wiped with 70% ethanol, and air-dried to minimize contamination. After the sediment was collected, the overlying seawater was carefully removed using sterile siphons to avoid disturbance of the sediment surface. At each station, nodule abundance and seafloor coverage were visually estimated, and sediment pH was measured (Orion Star, Thermo Fisher Scientific, Beverly, MA, USA) in situ.

Polymetallic nodule abundance was primarily determined using a direct measurement method. After polymetallic nodule samples were collected with a box corer, they were weighed using a balance with a precision of 1 g. Nodule abundance at each station was then calculated by dividing the total nodule mass by the sampling area of the box corer opening. Polymetallic nodule coverage at each station within the study area was determined by a white plastic quadrat measuring 50 cm × 50 cm, which was subdivided into 625 equal grids, with a minimum grid size of 2 cm × 2 cm. The collected polymetallic nodules were evenly spread within the quadrat with minimal overlap and without large gaps. The area occupied by the nodules within the quadrat was recorded, and nodule coverage was calculated by dividing the occupied area by the total quadrat area (0.25 m^2^).

After polymetallic nodules were collected using sterile gloves, vertical subsampling of the sediment column was immediately conducted on deck to minimize contamination. Before collecting each layer, the tools were sterilized with 70% ethanol to avoid contamination. The top 0–10 cm sediment samples of five sites were taken from the box cores using a push core with a diameter of 7 cm; two surface horizons—0–5 cm and 5–10 cm below the seafloor—were collected from each station and transferred into sterile 50 mL centrifuge tubes. All sediment subsamples were stored at −80 °C until further DNA extraction and geochemical analyses.

### 2.2. DNA Extraction and Amplicon Sequencing Analysis

Sediment DNA was extracted using the Soil FastDNA SPIN kit (MP Biomedicals, Solana Beach, CA, USA) following the procedures described by Tang et al. [36]. To enhance DNA yield from the sediment samples, DNA was concurrently extracted in triplicate from each of the three samples. Subsequently, total DNA was eluted from the triplicate samples using 60 µL of elution buffer. The concentration and purity of the extracted DNA were assessed using a Nanodrop spectrophotometer (Nanodrop 200, Thermo Fisher Scientific, Waltham, MA, USA). The primers employed for PCR amplification of the different taxonomic groups are provided in Table 1. Afterwards, paired-end sequencing was performed on the Illumina MiSeq PE250 platform (Illumina, San Diego, CA, USA) at Shanghai Meiji Bioengineering Co., Ltd., Shanghai, China.

### 2.3. Sequencing Data Processing and Downstream Analyses

Raw paired-end sequences were first processed using Trimmomatic for adapter removal and quality control [40], followed by primer removal. High-quality sequences were then imported into DADA2 (version 1.16) for quality filtering, denoising, error correction, and chimera removal to generate precise ASVs (Amplicon Sequence Variants). Taxonomic assignment was performed using QIIME 2’s classify-sklearn (Naive Bayes) classifier with a confidence threshold of 0.7, based on the SILVA 138.1 database for 16S rRNA and the PR2 database (version 5.0.0) for 18S rRNA. The unjoined raw paired-end 16S and 18S rRNA gene sequence data have been deposited in the NCBI Sequence Read Archive (SRA) under BioProject ID PRJNA1358054. The table of taxonomic composition at the ASV level is provided in Taxon_ASV_BPC (Supplementary Table S1).

From NCBI (BioProject ID PRJNA281530, SRA ID SRP057408), 16S rRNA and 18S rRNA sequence raw datasets from five stations of the MV1313 cruise (October 2013) in the UK-1 mining area of the Clarion-Clipperton Zone, collected aboard R/V Melville [19], were downloaded. For the UK-1 dataset, DADA2 could not be used for quality control due to data quality issues. Therefore, raw reads were imported into QIIME 2 (version 2024.2) and quality-filtered using the q-score method, including removal of ambiguous bases, trimming of low-quality regions, and discarding of short sequences. The filtered reads were then dereplicated using VSEARCH (version 2.30.0) to remove identical sequences. Subsequently, OTUs (operational taxonomic units) were generated by de novo clustering of the dereplicated sequences at 97% similarity. Representative sequences and the corresponding OTU table were taxonomically annotated using QIIME 2’s classify-sklearn plugin, which applies a Naive Bayes classifier trained on the same reference database with a confidence threshold of 0.7. The use of an identical database release and taxonomy files ensured consistency and reproducibility between sequence processing and downstream taxonomic assignment. Due to recent advances in microbial taxonomy, many phyla have been reclassified; for example, the formerly independent phylum Thaumarchaeota has been reassigned under the class Nitrososphaeria of the phylum Crenarchaeota in the latest SILVA 138 database. Thus, the taxonomic annotations in this study differed from those in the original publication.

To standardize the dataset, sequence data from the UK-1 at depths of 0–5 cm, 5–6 cm, 6–8 cm, and 8–10 cm were merged into two composite layers (0–6 cm and 6–10 cm) for subsequent analyses.

### 2.4. Statistical Analyses

To assess differences in bacterial, archaeal, and fungal community structures among stations in the western Pacific nodule field, Bray–Curtis distance matrices were calculated using the vegdist function from the vegan (version 2.7.1) inR (version 4.5.0). Principal coordinates analysis (PCoA) was then performed using functions in vegan to visualize community dissimilarities.

Co-occurrence networks were constructed based on Spearman correlation matrices using the Hmisc R package 4.5.0, and network topological properties were calculated. Correlations with |ρ| > 0.8 and *p* < 0.005 were visualized in Gephi (version 0.10.1) as co-occurrence networks. “Hub nodes” were defined as nodes with the highest degree within each network.

The taxonomic normalized stochasticity ratio (tNST) was calculated using the NST R package to assess the relative importance of deterministic and stochastic processes in the assembly of bacterial, archaeal, and fungal communities across different environments [41]. The tNST values range from 0 to 1, with 0.5 as the threshold. Communities with tNST values predominantly above 0.5 are considered to be mainly governed by stochastic processes, whereas communities with NST values predominantly below 0.5 are considered to be primarily shaped by deterministic processes.

### 2.5. Measurement of TOC, TN, and Stable Isotope Composition in Sediment Samples

Approximately 1 g of freeze-dried biological or sediment samples was ground prior to analysis. An appropriate amount of powdered samples (20–30 mg for sediments) with tin capsules (5 × 9 mm or 4 × 6 mm) was weighed separately, with acidification only for TOC (Total organic carbon) samples. TOC, TN (Total nitrogen), and the stable isotope ratios (δ^13^C and δ^15^N) were determined using an elemental analyzer coupled with an isotope-ratio mass spectrometer (EA-IRMS; Vario EL cube elemental analyzer coupled with IsoPrime 100 IRMS, Elementar GmbH, Langenselbold, Hesse, Germany and Elementar UK Ltd, Manchester, United Kingdom) at the Analysis and Test Center, Third Institute of Oceanography, Ministry of Natural Resources of China, Xiamen, Fujian, China. Laboratory reference standards were simultaneously analyzed to ensure accuracy, including Acetanilide #1 (δ^13^C = −29.53‰, δ^15^N = 1.18‰).

TOC samples were combusted at approximately 1020 °C in an elemental analyzer to produce CO_2_. The coupled isotope-ratio mass spectrometer measured the ^13^C/^12^C ratio of CO_2_, which was then compared to an international standard (Pee Dee Belemnite, PDB) to calculate the δ^13^C values of the samples. The analytical precision was ±0.2‰ for δ^13^C. TN samples were similarly combusted to produce N_2_, and the ^15^N/^14^N ratio of N_2_ was measured and compared to the international atmospheric N_2_ standard (Atm-N_2_) to determine δ^15^N values. The analytical precision was ±0.25‰ for δ^15^N.

## 3. Results

### 3.1. Microbial Community Structure and Distribution Patterns in the Western Pacific Polymetallic Nodule Field

#### 3.1.1. Microbial Community Composition

The number of sequence and ASVs obtained by 16S rRNA sequencing for bacteria and archaea, and by 18S rRNA sequencing for eukaryotes are summarized in Table 2.

Samples BC26_5_10 for bacteria and BC20_5_10 for eukaryotes were excluded from subsequent analyses due to the low concentration of DNA. Overall, the 0–5 cm layer yielded more ASVs than the 5–10 cm layer. The effective ASV counts for bacteria, archaea, and eukaryotes were 17,457, 1371, and 5875, respectively. Fungal ASVs were subsequently extracted from the eukaryotic dataset for further analyses.

**Figure 2:**
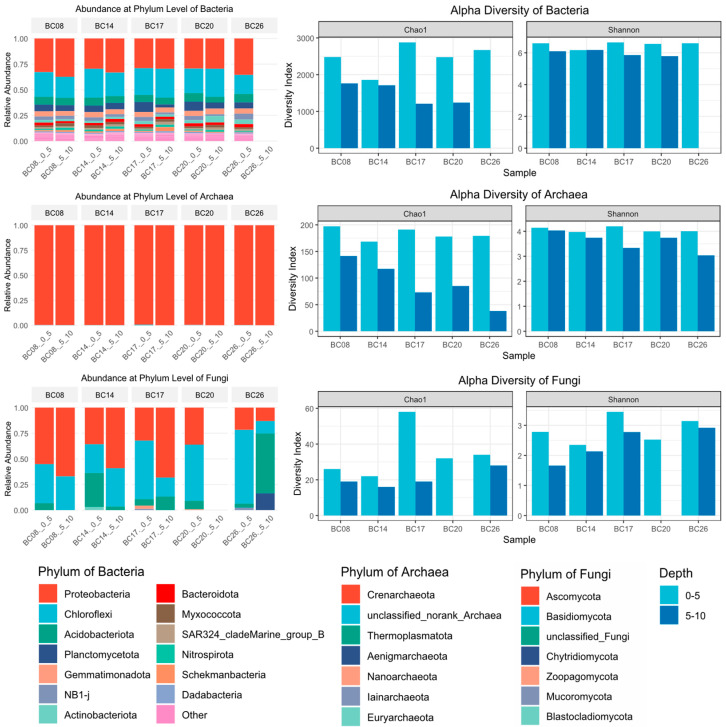
The phylum-level composition of microbial communities in the BPC of the western Pacific.

Figure 2 shows the phylum-level composition of microbial communities in the BPC of the western Pacific. Overall, the phylum composition of bacteria, archaea, and fungi was broadly similar across different stations and sediment depths. Based on Chao1 and Shannon indices, α-diversity of bacteria, archaea, and fungi was generally higher in surface sediments compared to that in deeper layers. Moreover, bacteria displayed the highest diversity indices, followed by archaea, while fungi exhibited the lowest. Shannon indices further indicated that microbial communities in the 0–5 cm layer were not only richer in species but also more evenly distributed. However, the larger variation in Shannon indices of Fungi in surface samples suggests the higher within-community heterogeneity among stations.

In the sediments of the BPC, bacterial communities were predominantly composed of Proteobacteria, Chloroflexi, Acidobacteriota, Planctomycetota, and Gemmatimonadota. At the phylum level, bacterial community structures were similar across different stations, but vertical distribution patterns were variable. Within the same station, the relative abundance of Proteobacteria in the 0–5 cm layer was generally lower than that in 5–10 cm layer, whereas Planctomycetota exhibited the opposite trend. Archaeal communities were overwhelmingly dominated by Crenarchaeota, accounting for over 99% of sequences, with all other phyla detected at very low abundances. For further details, archaeal taxonomic classification at the genus level is provided in Supplementary Figure S1. Fungal communities showed some variation in phylum-level composition among stations. At BC08, BC14, BC17, and BC20, the major phyla were Ascomycotina and Basidiomycota. In contrast, at BC26_5_10, a unique, unclassified fungal phylum was detected in deep-sea sediments with relative abundance of Chytridiomycota higher than at other stations.

**Figure 3:**
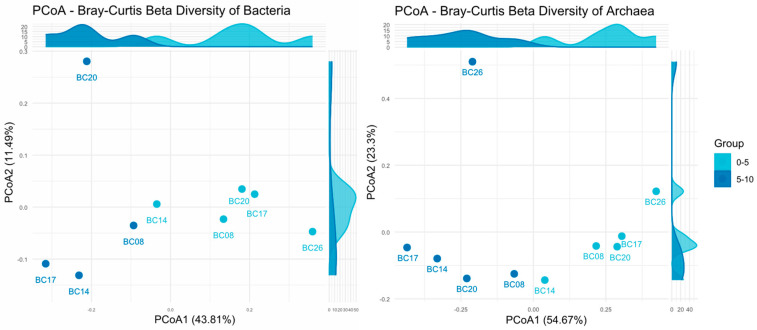
β-Diversity of Microbial Communities in Sediments from the Western Pacific Polymetallic Nodule Field.

The results of principal coordinates analysis (PCoA) for bacteria and archaea based on Bray–Curtis distances were shown in Figure 3. PCoA was not conducted for fungal communities, as the corresponding p-values were all greater than 0.05, indicating no significant differences.

The β-diversity of bacterial communities in the western Pacific sediments (PERMANOVA, R^2^ = 0.35, *p* = 0.018) was captured well by PCoA, with PCoA1 and PCoA2 explaining 43.81% and 11.49% of the total variation, respectively. Archaeal β-diversity showed a similar pattern (PERMANOVA, R^2^ = 0.45, *p* = 0.009), but with higher explanatory power along both axes: PCoA1 and PCoA2 accounted for 54.67% and 23.3% of the variation, respectively.

Bacterial and archaeal β-diversity distributions showed similar patterns, with samples in the 0–5 cm layer clustering more closely than those in the 5–10 cm layer, indicating greater similarity among surface samples. Moreover, similarities among different stations at the same depth were higher than those of different depths within the same station. β-diversity patterns of deep-layer samples were more closely followed geographic distribution gradients compared to surface samples.

#### 3.1.2. Correlations Between Microbial Communities and Environmental Factors

**Figure 4:**
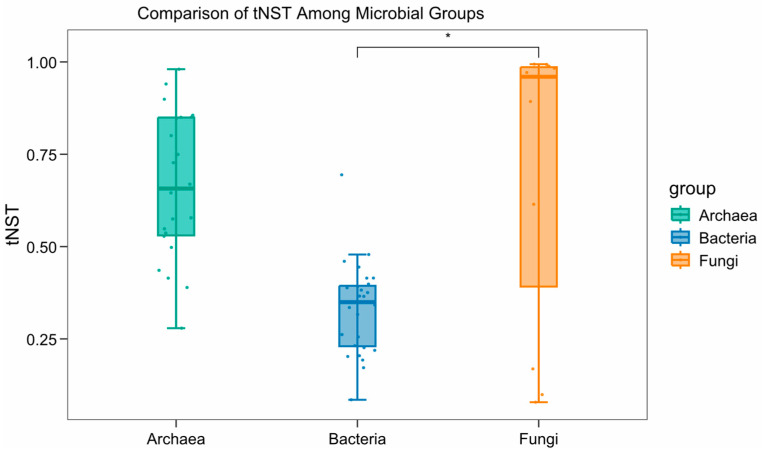
Microbial Community Assembly Processes in Benthic Sediments of the Western Pacific Polymetallic Nodule Field. * *p* < 0.05.

tNST analysis revealed that the assembly of microbial communities was more strongly influenced by environmental factors than by stochastic processes (Figure 4). Bacteria exhibited median tNST values of 0.25–0.35, indicating dominance of deterministic processes driven by environmental filtering. Archaea showed median tNST values around 0.75–0.80, suggesting stochastic processes predominated, while fungi had median tNST values close to 1 with the highest dispersion, reflecting a broad range from strongly deterministic to strongly stochastic assembly across different sampling sites. Pairwise comparisons indicated that fungal tNST values were significantly higher than those of bacteria (*p* < 0.05), suggesting that bacterial communities were most influenced by environmental factors, whereas fungal communities were least affected.

**Figure 5:**
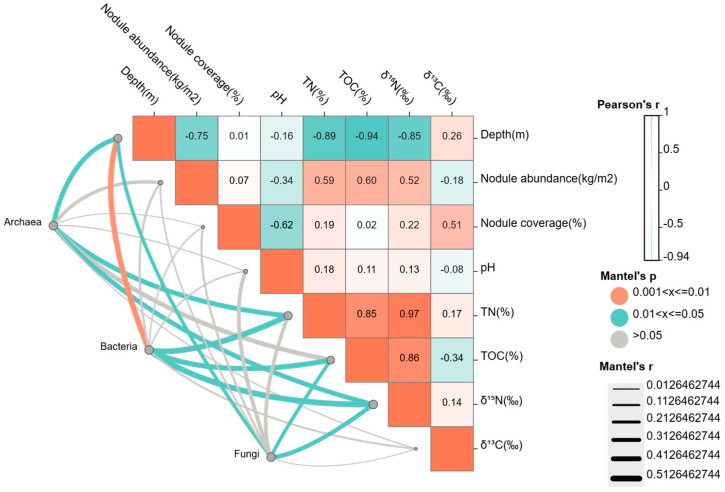
Correlations between Microbial Communities and Environmental Factors in Benthic Sediments of the Western Pacific Polymetallic Nodule Field.

Mantel test results indicated that the community structures of bacteria, archaea, and fungi were significantly correlated with water depth, TOC, TN, and δ^15^N, whereas their correlations with nodule abundance, coverage, pH, and δ^13^C were weak and not significant (Figure 5). Further correlation analysis among environmental factors revealed that water depth showed significant negative correlations with TOC, TN, δ^15^N, and nodule abundance. Both nodule abundance and coverage were negatively correlated with pH, while TN exhibited significant positive correlations with δ^15^N and TOC. These findings suggest that the major environmental factors in the study area are tightly coupled and collectively shape the spatial distribution patterns of sediment microbial communities.

Different microbial groups exhibited distinct response patterns to sedimentary environmental variables. Bacterial communities showed stronger responses to environmental factors compared with archaeal and fungal communities, displaying a significant negative correlation with water depth (Mantel test, R^2^ = −0.49, *p* = 0.008), and significant positive correlations with TOC, TN, and δ^15^N. Archaeal communities were significantly correlated with water depth, TN, and δ^15^N, suggesting that archaea may act as major participants in nitrogen cycling within the nodule field. Fungal communities were also correlated with water depth, TOC, and δ^15^N. However, the weak correlations between fungi and environmental factors imply that the spatial distribution of fungi may not only rely on localized water depth or organic matter distribution but also be controlled by other regional environmental factors.

### 3.2. Microbial Community Differences Between the Eastern and Western Pacific Polymetallic Nodule Fields

#### 3.2.1. Compositional Differences of Microbial Communities in the Eastern and Western Pacific Polymetallic Nodule Fields

**Figure 6:**
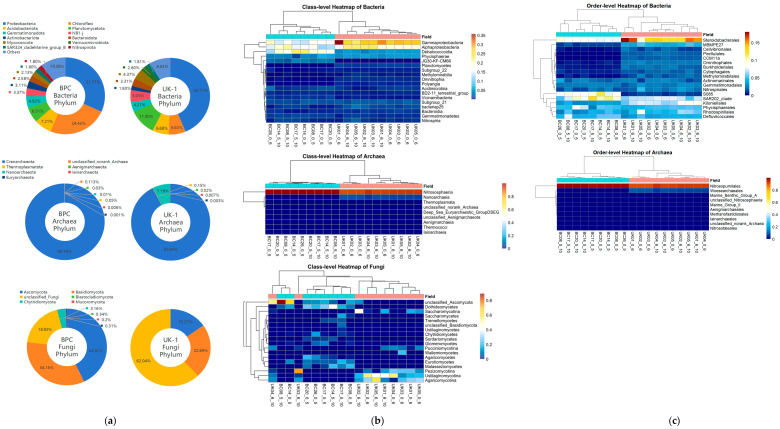
Comparison of microbial community composition at the phylum (**a**), class (**b**), and order (**c**) levels in sediments from polymetallic nodule areas of the western and eastern Pacific, including a hierarchical clustering heatmap at the class and order levels.

Comparative and clustering analyses on the microbial community structure between the eastern Pacific UK-1 and the western Pacific BPC in this study are shown in Figure 6.

In both nodule fields, the bacterial community was overall dominated by phyla such as Proteobacteria, Chloroflexi, and Planctomycetota. Among these, Chloroflexi was more prominent in the BPC, whereas Proteobacteria and Planctomycetota were relatively predominant in the UK-1. At the class level, Alphaproteobacteria and Gammaproteobacteria within Proteobacteria were predominant in both fields, with markedly higher abundances in the UK-1 compared to the BPC. In contrast, Dehalococcoidia and JG30-KF-CM66 within Chloroflexi showed higher relative abundances in the BPC than in the UK-1. Phycisphaerae (Planctomycetota) and Subgroup_21 (Acidobacteriota) were also relatively abundant in both fields, with comparable relative abundances between them. At the order level, the dominant taxa in BPC were S085 and SAR202_clade (Chloroflexi–Dehalococcoidia), whereas in UK-1, taxa were overwhelmingly concentrated in Steroidobacterales (Proteobacteria–Gammaproteobacteria), which was also relatively abundant in BPC. The divergence in bacterial community composition between the two nodule fields became increasingly evident as the analysis delved into finer taxonomic levels.

At the phylum level of archaea, Crenarchaeota represented the overwhelmingly dominant archaeal phylum in both the BPC and the UK-1. In the BPC, archaeal groups other than Crenarchaeota accounted for only 0.21%, whereas in the UK-1, Nanoarchaeota was the only other phylum exhibiting relatively high abundance. At the class level, a similar distribution pattern was observed: Nitrososphaeria, a class within Crenarchaeota, was likewise overwhelmingly dominant in the archaeal communities. In the UK-1, the relative abundance of Nanoarchaeia, a class within Nanoarchaeota, was also higher than that in the BPC. At the order level, the corresponding Nitrosopumilales (Crenarchaeota–Nitrososphaeria) was the dominant order in both fields, whereas Woesearchaeales (Nanoarchaeota–Nanoarchaeia) was relatively more abundant in UK-1.

The microbial communities in the eastern and western Pacific nodule fields exhibited certain similarities but also some differences in both phylum and class-level (Figure 6). The microbial assemblages in the BPC and UK-1 formed distinct clusters, indicating substantial differences in community structure between the two nodule fields.

Fungi were primarily represented by the phyla Ascomycota and Basidiomycota in both fields, with a relatively higher proportion of unclassified fungi observed in the UK-1. However, the fungal community composition exhibited pronounced spatial heterogeneity at the class level, with differences in dominant taxa between the two nodule fields being even more pronounced than those observed for bacteria and archaea. At the class level in the BPC, relatively higher abundances were observed for unclassified_Ascomycota, Dothideomycetes, and Eurotiomycetes within Ascomycota, as well as Malasseziomycetes (Basidiomycota). In the UK-1, the fungal community was mainly composed of Ustilaginomycotina and Agaricomycotina within Basidiomycota, and Pezizomycotina (Ascomycota). In the UK-1 area, fungal sequences could not be reliably classified at the order level, preventing the construction of meaningful order-level community profiles and precluding further comparisons at this taxonomic level.

#### 3.2.2. Differences in Microbial Co-Occurrence Networks

**Figure 7:**
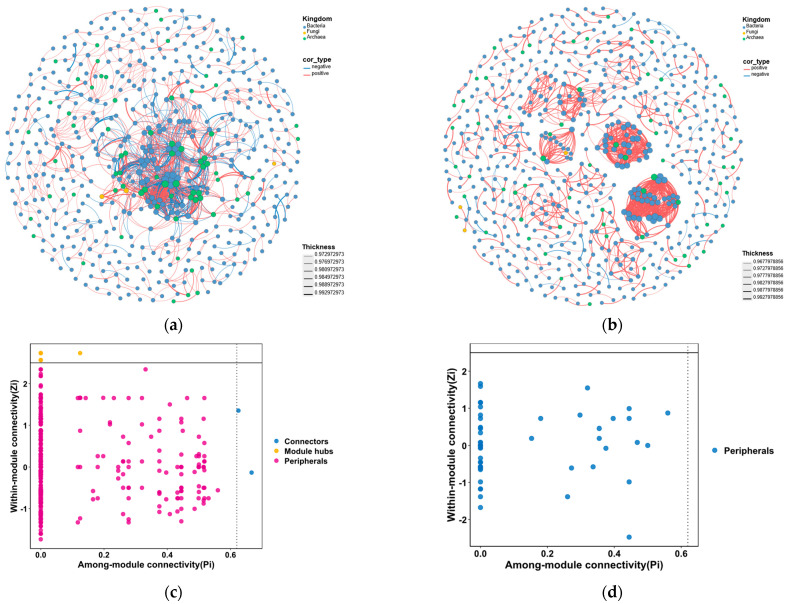
Co-occurrence Networks of Microbial Communities in Sediments of the Pacific Polymetallic Nodule Fields with Zi and Pi Analysis: (**a**) Networks of BPC; (**b**) Networks of UK-1; (**c**) Zi and Pi Analysis of BPC; and (**d**) Zi and Pi Analysis of UK-1.

Co-occurrence network analysis based on Spearman rank correlations (|ρ| > 0.8, *p* < 0.005) was applied and visualized to describe the co-occurrence patterns among bacteria, fungi, and archaea groups in the sediments of eastern and western Pacific nodule fields, respectively (Figure 7), the topological indices of the microbial co-occurrence networks are shown in Table 3.

Under the same correlation threshold, the network of BPC exhibited fewer nodes but a higher number of edges compared with the network of UK-1, while the latter displayed more pronounced modularity (Figure 7). These patterns indicated significant differences in community complexity and interaction characteristics between the two nodule fields. In the network of BPC (a), a highly connected core network was apparent, suggesting that certain species may engage in more extensive symbiotic or cooperative interactions, highlighting the roles of key taxa. Additionally, the higher network density and node connectivity reflected greater overall connectivity and tighter interactions among microbial communities. However, the relatively lower modularity and clustering coefficient suggest that the system may be more sensitive to perturbations of key nodes, with lower ecological resilience in the BPC. In contrast, the network of UK-1 (b) with higher modularity and clustering coefficient but fewer edges reflected a more dispersed community structure with stronger system stability and resilience, where interactions among taxa were likely dominated by localized mutualistic or metabolic complementarity relationships.

The Pi-Zi analysis further indicated that, compared with the western Pacific (c), the eastern Pacific network (d) contained almost no module hubs (nodes connecting multiple modules) and very few connectors (highly connected nodes within modules), further confirming that microbial interactions in the eastern Pacific were weaker than those in the western Pacific. In both mining areas, bacterial associations, as well as those between bacteria and archaea, were relatively strong, whereas associations involving fungi with either bacteria or archaea were comparatively weak. Furthermore, both connectors and module hubs were predominantly affiliated with bacterial taxa.

## 4. Discussion

### 4.1. Response of Microbial Community Structure to Environment in the Western Pacific Polymetallic Nodule Field

Based on the results, the microbial communities in the sediments of the western Pacific BPC were characterized by a higher diversity in surface sediments in comparison with that in deeper layers. As sediments within the 0–5 cm layer were influenced by bottom water, this condition may favor the microbial colonization and the formation of stable biofilms on surface sediment, but deeper sediment layers represent relatively stable anoxic environments [42]. These differences in oxygen availability may lead to variations in other factors, such as TOC and TN. Spatial heterogeneity and dispersal limitation likely contributed to the observed variations in α- and β-diversity of bacterial and archaeal communities between the 0–5 cm and 5–10 cm sediment layers.

Mantel test results confirmed that microbial taxa differed in their responses to environmental factors. In particular, bacteria and archaea were more strongly influenced by environmental variation, whereas fungi showed relatively weaker overall correlations with environmental parameters. This pattern was further supported by tNST analysis and was consistent with the “size-plasticity” hypothesis, which suggested that smaller organisms are more strongly affected by species sorting rather than dispersal limitation [43]. Consistently, bacterial and archaeal communities were more strongly influenced by water depth, TN, TOC, and δ^15^N than fungal communities.

Although the presence of polymetallic nodules could increase the concentrations of heavy metal ions in sediments, previous studies have shown that microbial taxa in the nodule field possess various resistance genes to cope with environmental metal stress. For example, microbes may maintain metal homeostasis through regulated ion transport systems. Complex interactions among sulfur-metabolizing bacteria may enable the formation of metal sulfides, allowing adaptation to elevated metal concentrations [44]. This may explain why the variations in nodule abundance and coverage did not affect the microbial community structure significantly.

### 4.2. Factors Contributing to the Differences in Microbial Community Structure Between the Eastern and Western Pacific Polymetallic Nodule Fields

Microbial communities in the benthic sediments of both the eastern and western Pacific nodule fields were diverse and complex. Comparative analyses indicated that microbial communities in the eastern and western Pacific nodule fields shared certain similarities, while also displaying distinct differences at different levels.

At the phylum level, the most pronounced differences were observed for the bacterial lineages Proteobacteria, Chloroflexi, and Planctomycetota; the archaeal lineage Nanoarchaeota; and the fungal lineages Ascomycota and Basidiomycota, all of which exhibited relatively higher abundances in the BPC. Chloroflexi in deep-sea sediments exhibit a heterotrophic lifestyle with the metabolic potential to degrade a wide range of organic, sulfur, and halogenated compounds, including recalcitrant organic matter, while also being able to synthesize energy storage compounds and regulate their metabolism in response to nutrient availability, suggesting a “feast-or-famine” strategy in hadal environments [45], this suggests that the environment in the BPC is characterized by nutrient instability. Planctomycetes are primarily associated with particles, microbial mats, and biofilms [46], and can constitute up to 22% of the bacterial community in marine snow [47], their higher relative abundance in UK-1 may indicate a greater contribution of particle-associated or sinking organic matter to this site.

A unique dominant archaeal phylum in the eastern Pacific UK-1 is Nanoarchaeota, belonging to the DPANN superphylum. This group primarily lives as epibionts on the surface of marine hyperthermophilic archaea (*Ignicoccus hospitalis*) and possesses metabolic pathways and ecological functions adapted to deep-sea hydrothermal environments [48,49]. Nanoarchaeota and Crenarchaeota are methanogenic archaea, the main producers of biogenic methane, capable of utilizing three metabolic pathways: the hydrogenotrophic pathway (based on CO_2_ and H_2_), the acetoclastic pathway, and the methylotrophic pathway (based on methyl compounds) [50,51].

Current evidence indicates that Ascomycota dominate marine fungal communities and exhibit strong protease and chitinase activity, particularly in polar waters, whereas Basidiomycota are more active in non-polar, warmer regions and contribute disproportionately to carbohydrate-active enzymes involved in complex polysaccharide degradation [52].

At the class level, the microbial communities in both nodule fields are dominated by the bacterial groups Gammaproteobacteria, Alphaproteobacteria, and Dehalococcoidia, as well as the archaeal class Nitrososphaeria. In contrast, fungal communities exhibit almost no class-level similarity between the two fields. Consistent with previous research, Gammaproteobacteria exhibit versatile and opportunistic metabolic capabilities, enabling the transformation of organic carbon and sulfur compounds and exerting widespread ecological impacts across the global deep ocean [53]. Dehalococcoidia exhibit diverse metabolic capacities—most notably the reductive dehalogenation of natural organohalogens and potential links to C, S, and N cycling—indicating important roles in halogen turnover and carbon processing in the deep-sea environment [54]. Although both the western Pacific BPC and eastern Pacific UK-1 are deep-sea polymetallic nodule fields with broadly similar environmental characteristics, notable differences between them may explain the observed variations in microbial communities.

First, differences in microbial diversity and community structure are closely linked to organic matter (OM) content, which is directly influenced by variations in primary productivity in the overlying waters, as well as the water depth and OMZs of the two nodule fields. Based on global satellite-derived surface chlorophyll data, chlorophyll-a concentrations in UK-1 were mainly within the range of 0.11–0.13 mg/m^3^, while that from the M2 seamount region in the western Pacific indicate an average concentration of only 0.094 mg/m^3^ [55]. This east–west gradient in surface chlorophyll-a is largely driven by the westward-flowing North Equatorial Current (NEC), resulting in a strong gradient of chlorophyll concentrations from east to west [56]. Moreover, the UK-1 is generally located at water depths around 4000 m, whereas the samples collected from BPC were no less than 5000 m in depth. This depth difference likely contributes directly to organic sequestration, resulting in relatively higher TOC and TN levels in the UK-1 sediments than in other CCZ areas of the eastern Pacific [29,30].

In addition, oceanic oxygen minimum zones (OMZs) play a critical role in regulating the distribution of organic carbon in sediments, as the attenuation of particulate organic carbon (POC) flux with depth is weaker in OMZ waters when compared with well-oxygenated waters [57,58]. When microbial communities in the water column shift from aerobic to anaerobic metabolism under low-oxygen conditions, the decomposition of organic matter slowed, allowing a greater fraction to reach the deep-sea substrate. The OMZs of the tropical Pacific are mainly concentrated in the eastern Pacific, where oxygen levels are lower than those in the western Pacific [59]. Consequently, POC input to benthic sediments as a whole in the eastern Pacific nodule field is relatively higher than that in the western Pacific nodule field. This may explain the observed differences in microbial community composition and diversity between the two fields.

Secondly, differences in sedimentary environment also exist in the nodule coverage between the two fields. For example, nodule abundance in UK-1 reaches up to 57 kg/m^2^ [31], which is higher than the 25–40 kg/m^2^ observed in the BPC. Although nodule abundance was not correlated with the microbial community variation in the BPC, the properties of nodules may be important factors influencing regional microbial distribution [29]. As shown in previous studies, nodules in the eastern Pacific are enriched in Mn, Cu, and Ni, whereas those in the western Pacific contain higher levels of Fe, Ti, and Co [60]. Based on Mn/Fe ratios, nodules in the western Pacific exhibit a clear hydrogenetic signature [61,62], whereas those in the eastern Pacific generally display a mixed diagenetic-hydrogenetic origin, with diagenetic contributions predominant [60]. Furthermore, hydrothermal precipitates are also an important source of metals in polymetallic nodules [63], tracer experiments have shown that in the eastern Pacific, hydrothermal plumes can transport He-3, dissolved Fe, colloidal Fe-Mn, and some rare earth elements, along with animal larvae, from hydrothermal source regions to the basin by means of neutrally buoyant plumes, lateral jets, and vortices [64,65].

Notably, the raw data from the UK-1 mining area (published in 2016) could not be used to generate ASV data because sequencing quality limitations prevented the application of DADA2 denoising, leading to differences in data processing workflows compared with the Western Pacific samples. Previous studies based on soil, rhizosphere, and human microbiome datasets have shown that OTU- and ASV-based methods produce generally similar biological signals [66,67]. OTU clustering tends to substantially reduce species diversity indices and exhibits systematic biases in dominance and evenness compared with ASV data [68,69]. Instead, the overall community composition at the phylum and family levels are highly consistent [66,70]. Accordingly, direct comparisons on the biodiversity indices between the two mining areas were not conducted in this study. The overall observed differences in bacterial, archaeal, and fungal community composition reflected the specific biogeochemistry of benthic sediments in the eastern and western Pacific polymetallic nodule fields, as well as regional differences in ecological responses.

### 4.3. Potential Stability of Microbial Community Structure in the Eastern and Western Pacific Polymetallic Nodule Fields

Previous study suggested that archaea and fungi contribute significantly to the stability of bacterial communities across different habitats, particularly under low-temperature conditions where their close associations with bacteria enhance the adaptability of microbial communities to cold stress, with the microbial network structure being primarily influenced by the nutrient contents in water and sediments [71]. As suggested by the Stress Gradient Hypothesis (SGH) [72], the balance between cooperative and competitive interactions shifts inversely along gradients of abiotic stress: cooperation is more common under high-stress conditions, whereas competition predominates under low-stress conditions. Negative correlations among nodes are relatively pronounced in the BPC, suggesting a low-stress condition under a more stable, low-nutrient environment, where competitive and antagonistic interactions among microbial taxa were more pronounced. In contrast, microbial networks in the eastern Pacific displayed a tighter inter-domain connectivity (higher modularity) and lower inter-taxa interaction, indicating a higher environmental stress owing to the variable water depth and organic matter input.

Karimi et al. [34] proposed linking microbial networks with ecological functions as a practical and quantitative approach to assess ecosystem quality and functionality, because they found that even when microbial diversity parameters remain stable, key network metrics such as connectivity, number of links, and average co-occurrence can respond to minor perturbations by 10–50%. Similarly, microbial co-occurrence networks are also found to be sensitive to disturbance, such as soil metal pollution, land-use types, and global warming [34,73]. Based on the results of this study, the network of BPC was more strongly influenced by key nodes and exhibits a tighter overall structure, whereas the network of BPC showed weaker connectivity among microbial taxa but higher modularity, indicating greater network stability in the sediments of UK-1. Under potential future disturbances such as surface sediment removal due to mining, benthic microbial communities in the UK-1 may exhibit higher resilience in comparison with those in the BPC.

The exploitation of polymetallic nodules is inevitably expected to exert varying degrees of environmental impact [74]. Because oxic manganese sediments and nodule particles are more effective at sequestering heavy metals than low-manganese sediments [75], removal of the upper 5–10 cm of deep-sea sediments during nodule mining may expose the oxic manganese layer and fragmented nodule fragments, substantially increasing the flux of trace metals from pore waters and nodule fragments, thereby causing short-term spikes in marine metal fluxes [76,77]. Under mining-induced environmental stress, co-occurrence networks can serve as functional indicators beyond community composition and diversity, reflecting micro-scale interactions among microbial taxa and their regulatory strategies for maintaining biodiversity [78]. They offer a promising approach for assessing the environmental impact of deep-sea mining on benthic microbial communities, as well as the potential for ecosystem recovery.

## 5. Conclusions

Based on analysis of community structure, diversity, and Mantel tests of environmental correlations for bacteria, archaea, and fungi in benthic sediments of the western Pacific BPC, and further comparative analyses with the eastern Pacific UK-1 at both community and co-occurrence network levels, the following conclusions were drawn:

1. The BPC exhibited relatively high biodiversity. Bacterial communities were dominated by Proteobacteria and Chloroflexi, while Crenarchaeota represented the absolute dominant archaeal group. Fungal communities showed considerable variability, with Ascomycotina and Basidiomycota being the predominant phyla at most sites.
2. Microbial groups displayed differential correlations with different environmental factors. Community composition was particularly strongly associated with water depth, TOC, TN, and δ^15^N.
3. The microbial communities in the eastern and western Pacific nodule fields exhibited certain similarities but also some differences at the phylum, class, and order levels, with the differences becoming increasingly pronounced at finer taxonomic resolutions. These differences were evident in: Chloroflexi were more prominent in the BPC, whereas Proteobacteria and Planctomycetota showed higher relative abundances in the UK-1, with differences becoming increasingly pronounced at the class and order levels within these phyla. Among archaea, Nanoarchaeota (Nanoarchaeia–Woesearchaeales) were more abundant in the UK-1 than in the BPC. For fungi, Ascomycota and Basidiomycota accounted for a larger proportion of the community in the BPC compared with the UK-1 and showed pronounced compositional differences at the class level.
4. Compared with UK-1, the BPC displayed distinct differences in co-occurrence patterns. The co-occurrence network in the BPC exhibited higher network density and node connectivity, whereas the UK-1 showed greater modularity and clustering coefficient, indicating that the microbial communities in the BPC had lower stability and resistance to disturbance relative to those in the UK-1 under future deep-sea mining disturbance.

## Figures and Tables

**Table 1:** The primers for bacterial and archaeal 16S rRNA and fungal 18S rRNA.

Domain	Primer	Target Region	Reference
Bacteria	338F (5′-ACTCCTACGGGAGGCAGCAG-3′)	16S rRNA V3–V4	[36]
	/ 806R (5′-GGACTACHVGGGTWTCTAAT-3′)		
Fungi	1380F (5′-CCCTGCCHTTTGTACACAC-3′)	18S rRNA V9	[37,38]
	/ 1510R (5′-CCTTCYGCAGGTTCACCTAC-3′)		
Archaea	524F10extF (5′-TGYCAGCCGCCGCGGTAA-3′)	16S rRNA V5–V6	[39]
	/ Arch958RmodR (5′-YCCGGCGTTGAVTCCAAT-3′)		

**Table 2:** Sequencing Information for Bacteria, Archaea, and Eukaryotes.

Sample_Info	Bacteria_ASV	Bacteria_Seq	Archaea_ASV	Archaea_Seq	Eukaryota_ASV	Eukaryota_Seq
BC08_0_5	2444	52776	197	24465	794	74291
BC08_5_10	1758	51828	142	25328	338	71976
BC14_0_5	1842	51828	170	25998	385	69240
BC14_5_10	1697	50696	117	22702	210	68070
BC17_0_5	2803	49063	191	18910	1419	81448
BC17_5_10	1205	51149	74	24208	261	43214
BC20_0_5	2433	47741	178	20886	1081	51451
BC20_5_10	1238	31566	85	23193	-	-
BC26_0_5	2623	52947	179	23664	1156	67722
BC26_5_10	-	-	38	53530	191	385918

**Table 3:** Topological Indices of Microbial Co-occurrence Networks in Sediments of the Pacific Polymetallic Nodule Fields.

	Edges Number	Positive Edges	Negative Edges	Nodes Number	Average Degree	Average Clustering Coefficient	Modularity	Network Diameter	Average Path Length	Density
BPC	1268	66%	34%	541	4.688	0.106	0.640	20.501	5.227	0.0087
UK	1008	96.5%	3.5%	632	3.180	0.277	0.876	7.839	1.691	0.0051

## Data Availability

The original contributions presented in this study are included in the article/Supplementary Materials. Further inquiries can be directed to the corresponding author.

## Supplementary Materials

The following supporting information can be downloaded at https://www.mdpi.com/article/10.3390/microorganisms14010103/s1: Figure S1: Supplementary Figure; Table S1: Taxon_ASV_BPC.
