# Supplementary figures and images for: Benthic Microbial Community Features and Environmental Correlates in the Northwest Pacific Polymetallic Nodule Field, with Comparative Analysis Across the Pacific
Source: Microorganisms. 2026 Jan 3;14(1):103. doi: 10.3390/microorganisms14010103 (PMC12844153; doi:10.3390/microorganisms14010103)

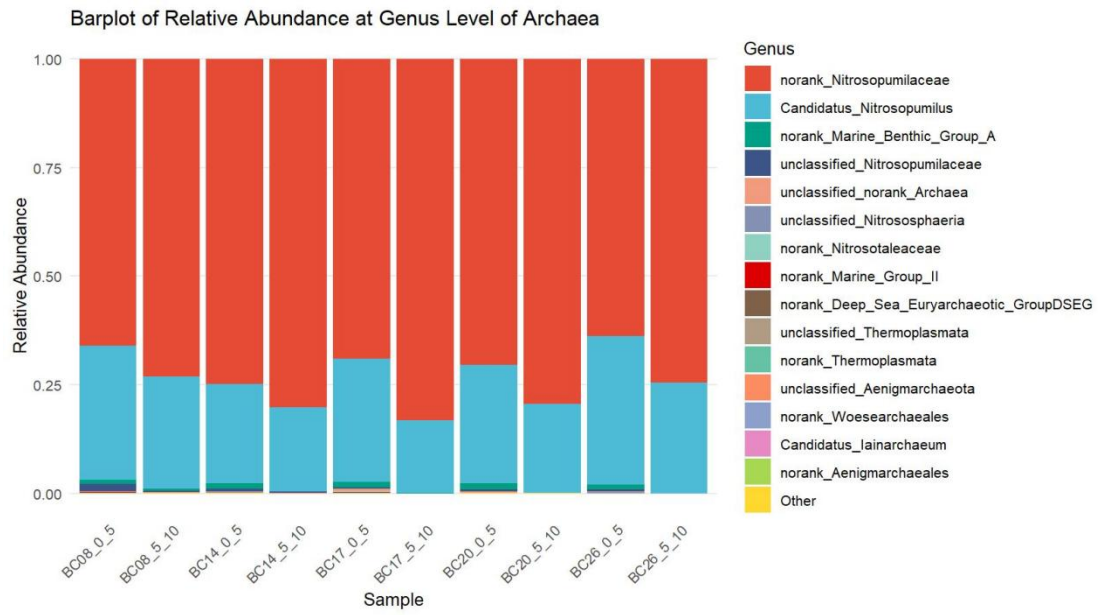

Supplementary Figure S1: Relative abundance of archaea at the genus level.

Supplement: Supplementary file 1 [file microorganisms-14-00103-s001.zip › Supplementary Figure S1.pdf]
